# Crystal structure of a 1:1 salt of 4-amino­benzoic acid (vitamin B_10_) with pyrazinoic acid

**DOI:** 10.1107/S2056989018016663

**Published:** 2018-11-30

**Authors:** K. V. Drozd, S. G. Arkhipov, E. V. Boldyreva, G. L. Perlovich

**Affiliations:** a G. A. Krestov Institute of Solution Chemistry of the Russian Academy of Sciences, 1, Academicheskaya, Ivanovo 153045, Russian Federation; b Novosibirsk State University, Pirogova str. 2, Novosibirsk, 630090, Russian Federation; c Institute of Solid State Chemistry and Mechanochemistry SB RAS, Kutateladze str. 18, Novosibirsk 630128, Russian Federation; d G. K. Boreskov Institute of Catalysis SB RAS, Laverentiev Ave. 5, Novosibirsk 630090, Russian Federation

**Keywords:** crystal structure, 4-amino­benzoic acid, pyrazinoic acid, salt, hydrogen bonding, melting point

## Abstract

The title 1:1 salt, C_7_H_8_NO_2_^+^·C_5_H_3_N_2_O_2_^−^ (systematic name: 4-carb­oxy­anilinium pyrazine-2-carboxyl­ate), was synthesized successfully by slow evaporation of a saturated solution from water–ethanol (1:1 *v*/*v*) mixture and characterized by X-ray diffraction (SCXRD, PXRD) and calorimetry (DSC). The crystal structure of the salt was solved and refined at 150 and 293 K. The salt crystallizes with one mol­ecule of 4-amino­benzoic acid (PABA) and one mol­ecule of pyrazinoic acid (POA) in the asymmetric unit. In the crystal, the PABA and POA mol­ecules are associated via COOH⋯N_arom_ heterosynthons, which are connected by N—H⋯O hydrogen bonds, creating zigzag chains. The chains are further linked by N—H⋯O hydrogen bonds and π–π stacking inter­actions along the *b* axis [centroid-to-centroid distances = 3.7377 (13) and 3.8034 (13) Å at 150 and 293 K, respectively] to form a layered three-dimensional structure.

## Abstract 2

The paper reports the crystal structure of novel salt of 4-amino­benzoic acid (Vitamin B_10_) with pyrazinoic acid.

## Chemical context   

4-Amino­benzoic acid (PABA) is known as vitamin B_10_ and is involved in the production of folic acid in bacteria (Chang & Hu, 1996 ▸; Akberova, 2002 ▸). It is used as an anti­bacterial (Richards *et al.*, 1995 ▸), anti-inflammatory (Flindt-Hansen & Ebbesen, 1991 ▸), anti­oxidant (Sirota *et al.*, 2017 ▸; Galbinur *et al.*, 2009 ▸), anti­coagulant (Stroeva *et al.*, 1999 ▸; Drozd *et al.*, 2000 ▸), or dermatologic agent (Rothman & Henningsen, 1947 ▸; Xavier *et al.*, 2006 ▸; Hanson *et al.*, 2006 ▸). Moreover, it is a building block used in the design of drug candidates and is frequently found as a structural moiety in drugs (Kluczyk *et al.*, 2002 ▸). PABA has been the subject of many scientific investigations, due not only to its pharmaceutical and biological properties, but also its ability to form various multi-component solid forms. PABA is a simple organic mol­ecule with two functional groups: amine and carboxyl. This makes it unique in its ability to form various hydrogen-bonded network structures (Athimoolam & Natarajan, 2007 ▸). Among all the multi-component crystals of PABA known to date, co-crystals and salts of PABA are especially numerous.
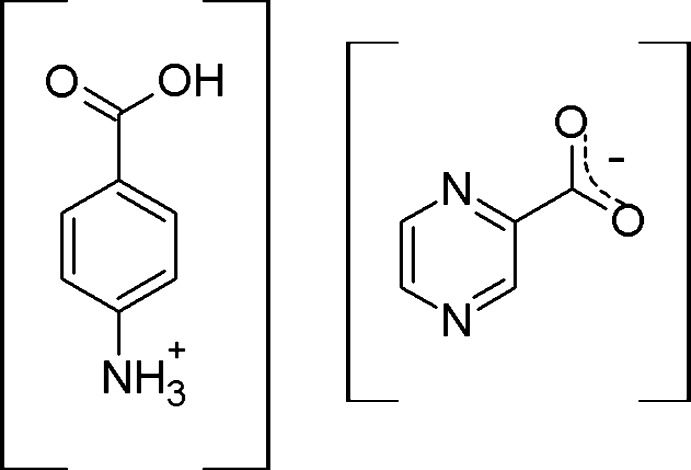


Today, the formation of either salts or co-crystals of APIs is one of the promising strategies to modify the solid-state properties of pharmaceutical compounds, such as solubility, bioavailability, stability, *etc.* (Shevchenko *et al.*, 2012 ▸; Perumalla & Sun, 2013 ▸; Manin *et al.*, 2018 ▸). The main difference between a salt and a co-crystal is in the position of a proton. A salt is formed if a proton is transferred from an acid to a base (Aakeröy *et al.*, 2007 ▸). Childs *et al.* (2007 ▸) and Cruz-Cabeza (2012 ▸) have noticed a linear correlation between Δp*K*_a_ [p*K*_a_(base) – p*K*_a_(acid)] of the starting compounds and the probability of the formation of either a salt or a co-crystal. It is assumed that a salt is expected to be formed if Δp*K*_a_ > 3 (Childs *et al.*, 2007 ▸) or Δp*K*_a_ > 4 (Cruz-Cabeza, 2012 ▸), whereas a co-crystal forms when Δp*K*_a_ < 0 (Childs *et al.*, 2007 ▸) or Δp*K*_a_ < −1 (Cruz-Cabeza, 2012 ▸). In the inter­mediate Δp*K*_a_ range, the nature of multi-component crystal is difficult to predict – a so called ‘salt–co-crystal continuum’ (Childs *et al.*, 2007 ▸; Hathwar *et al.*, 2010 ▸). Several examples have been documented where both a salt and a co-crystal could be formed by the same components from the same solutions under different crystallization conditions (Fu *et al.*, 2016 ▸; Losev & Boldyreva, 2018*a* ▸,*b* ▸). A co-crystal can also be converted into a salt in the solid state upon temperature variations (Grobelny *et al.*, 2011 ▸).

The present study reports the synthesis and crystallization of a novel salt of 4-amino­benzoic acid with pyrazinoic acid (pyrazine-2-carb­oxy­lic acid, POA), [PABA-POA], which was characterized using single crystal and powder X-ray diffraction (SCXRD, PXRD) and different scanning calorimetry (DSC).

## Elucidation of the multi-component crystal nature   

4-Amino­benzoic acid is an ampholyte mol­ecule with basic (–NH_2_) and acidic (–COOH) functional groups, and its p*K*_a_ values are 2.46 and 4.62 (Avdeef, 2017 ▸) respectively. Pyrazinoic acid is a weak acid with a p*K*_a_ of 2.9 (Zhang *et al.*, 1999 ▸). According to the Δp*K*_a_ of PABA and POA, the two-component crystal is within the range of the ‘salt–co-crystal continuum’. Both a salt and a co-crystal can be expected to crystallize.

The crystal structure of the title compound was solved and refined at 150 K (**Ia**) and 293 K (**Ib**). The nature of the crystal form (salt/co-crystal) was identified from the structural characteristics, namely the C—N bond length of PABA and the C—O bond lengths of the carb­oxy­lic/carboxyl­ate groups of PABA and POA at both temperatures to eliminate the possibility of salt–co-crystal transition. In a neutral pure PABA mol­ecule, the length of the C—N bond between the N atom of the amine group and the C atom of the benzene ring is *ca* 1.37–1.4 Å. In the title compound, the protonation of the PABA amine group results in a significantly longer C—N bond [1.455 (5) Å at 150 K and 1.467 (3) Å at 293 K]. To define the deprotonation site, the C—O bond lengths of both PABA and POA were compared. In a neutral carb­oxy­lic group, C—O is longer than C=O by 0.08 Å, or more. Deprotonation of a –COOH group leads to a decrease in this difference to 0.03 Å or less (Childs *et al.*, 2007 ▸; Chen *et al.*, 2012 ▸). In the title compound, the difference *d*(C—O) is 0.104 (6) or 0.102 (8) Å for PABA and 0.007 (6) or 0.012 (6) Å for POA at 150 K and 293 K, respectively, indicating deprotonation of the POA –COOH group and the formation of a salt.

## Structural commentary   

**Figure 1:**
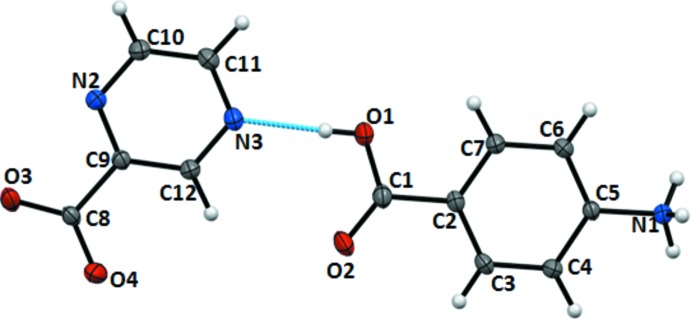
The asymmetric unit of the title compound at 150 K, with displacement ellipsoids drawn at the 50% probability level for non-H atoms. H atoms are shown as spheres of arbitrary radii.

The title compound crystallizes in the monoclinic non-centrosymmetric space group *Pc* with one mol­ecule of each component per asymmetric unit (Fig. 1 ▸). The carboxyl planes of PABA and POA are slightly twisted from the aromatic ring planes [2.76 (16) and 8.4 (2)° for **Ia**; 2.89 (19) and 9.2 (3)° for **Ib**], which is a characteristic feature found in almost all known multi-component complexes of both compounds. No phase transitions occur in the temperature range between 293 and 150 K.

## Supra­molecular features   

**Figure 2:**
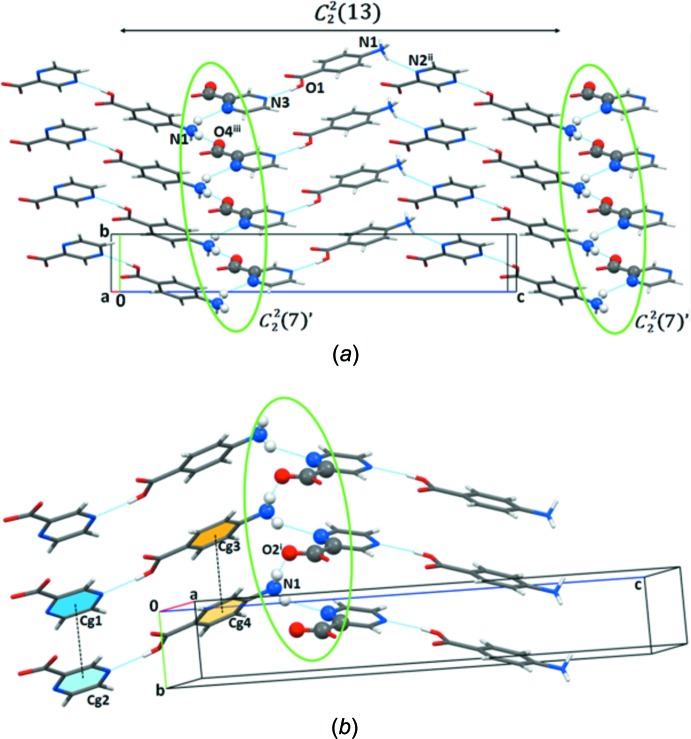
(*a*) The formation of zigzag 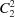
(13) chains through O1—H1⋯N3 and N1—H1*B*⋯N2^ii^ inter­actions joined by an N1—H1*C*⋯O4^iii^ hydrogen bond [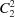
(7)’ chain motif] to generate the two-dimensional structure. (*b*) Layered arrangements of the salt via N1—H1*A*⋯O3^i^ inter­actions [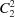
(7)’’ chain motif] and aromatic π–π stacking inter­actions (dotted black lines) to generate the three-dimensional structure. Symmetry codes are in Table 1 ▸.

In the crystal, the O1—H1⋯N3 hydrogen bond involving the carboxyl group of PABA and the pyridine one of POA forms an acid⋯pyridine heterosynthon (COOH⋯N_arom_, Tables 1 ▸ and 2 ▸). The neighboring two-component units are linked by N1—H1*B*⋯N2^ii^ hydrogen bonds, forming a zigzag 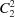
(13) chain motif. Adjacent chains are linked to each other via N1—H1*C*⋯O4^iii^ hydrogen bonds [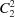
(7)’ chain motif] to form a 2D structure [Fig. 2 ▸(*a*)]. The crystal packing is stabilized by stacking of the parallel 2D structures along the *b*-axis direction through π–π inter­actions between neighboring benzene and pyrazine rings [*Cg*1⋯*Cg*2 = *Cg*3⋯*Cg*4 = 3.7377 (13) and 3.8034 (13) for **Ia** and **Ib**, respectively; *Cg*1 and *Cg*2 are centroids of the POA N2–C9 pyrazine ring, *Cg*3 and *Cg*4 are centroids of the PABA C2–C7 benzene ring], forming a 3D structure supported via N1—H1*A*⋯O3^i^ hydrogen bonds [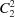
(7)’’ chain motif] [Fig. 2 ▸(*b*)].

## Thermal analysis   

**Figure 3:**
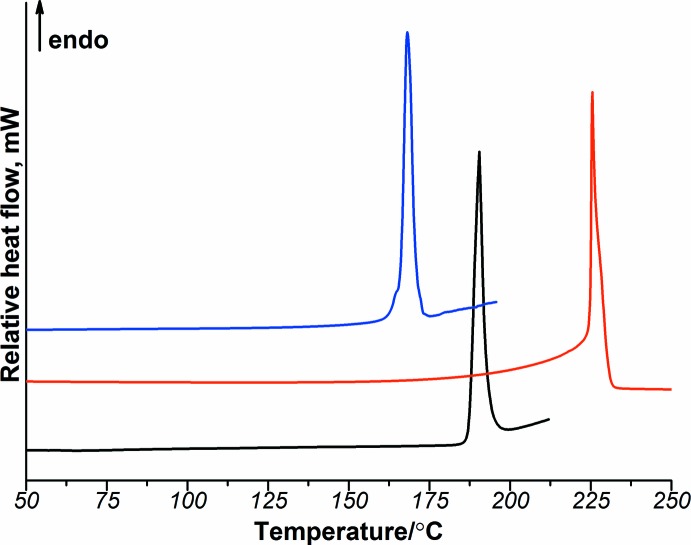
DSC curves of PABA (black), POA (red) and [PABA+POA] (blue).

The thermal behavior of the title compound was investigated by DSC techniques. The DSC curve [PABA+POA] is shown in Fig. 3 ▸. For a comparison, the DSC curves of the starting compounds are also plotted. PABA and POA show single endothermic peaks at 188.5 and 224.8°C, respectively. [PABA+POA] exhibits a sharp endothermic peak at 166.1°C. The melting temperature of the salt is *ca* 20 and 60°C lower than that of the starting compounds, suggesting the formation of a new crystalline phase. A single endothermic peak for the salt indicates that the solid state is homogeneous, and also suggests that there is no solvent in the crystal.

## Database survey   

A search of the Cambridge Structural Database (CSD version 5.39, May 2018 update; Groom *et al.*, 2016 ▸) for organic multi-component crystals (salts/co-crystals, their polymorphs and solvates) gave 88 structures for PABA and only five structures for POA. Analysis of the PABA crystal structures showed that the two most typical hydrogen-bonded motifs for them are: the acid⋯pyridine (COOH⋯N_arom_) heterosynthon as in the title compound and the acid⋯acid (COOH⋯COOH) homosynthon between PABA mol­ecules or PABA and conformer mol­ecules with carb­oxy­lic functional group.

## Synthesis and crystallization   

**Figure 4:**
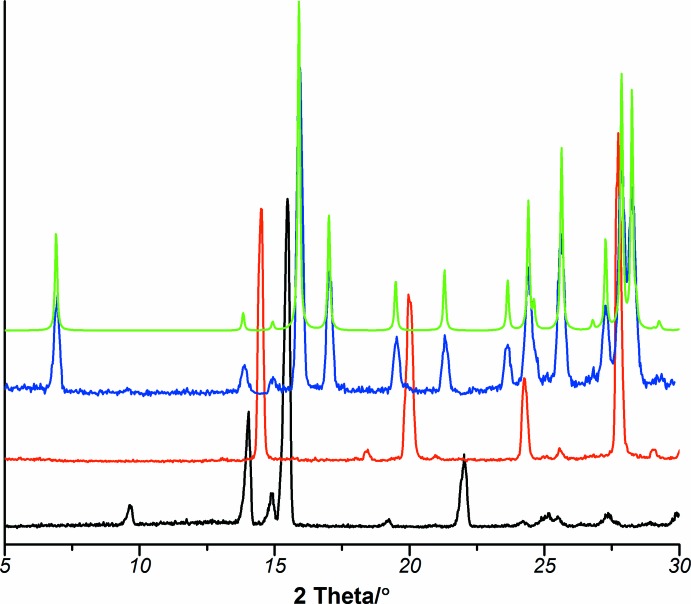
Comparison of the experimental PXRD patterns of [PABA+POA] prepared by liquid-assisted grinding (blue) of PABA (black) and POA (red) and calculated (green) using single-crystal X-ray diffraction data.

A commercial sample of PABA (Merck, 99%) was co-crystallized with POA (Acros organics, 99%) by either liquid-assisted grinding, or by slow evaporation from solution under ambient conditions. Single crystals of [PABA+POA] were grown at room temperature by slow evaporation of a water–ethanol (1:1 *v*/*v*) solution in a 1:1 stoichiometric ratio. The powder sample of the title compound for DSC analysis was obtained by liquid-assisted grinding of the physical mixture in the presence of ethanol using a planetary micro mill. The ground material was characterized using PXRD to verify the formation of a new phase by comparing the diffraction pattern with the powder pattern calculated based on the single crystal X-ray diffraction data obtained in this work (Fig. 4 ▸).

## Refinement   

Crystal data, data collection and structure refinement details are summarized in Table 3 ▸. The positions of all H atoms at 293 K were optimized geometrically and refined using a riding model, with the following assumptions and restraints: N—H = 0.89 Å, C—H = 0.93 Å and O—H = 0.82 Å with *U*_iso_(H) = 1.5*U*_eq_(O) for the hydroxyl groups, and 1.2*U*_eq_(C, N) otherwise. The positions of the H atoms at 150 K were refined freely in an isotropic approximation.

## Supplementary Material

Crystal structure: contains datablock(s) Ia, Ib. DOI: 10.1107/S2056989018016663/rz5246sup1.cif

Structure factors: contains datablock(s) Ia. DOI: 10.1107/S2056989018016663/rz5246Iasup4.hkl

Structure factors: contains datablock(s) Ib. DOI: 10.1107/S2056989018016663/rz5246Ibsup5.hkl

Supporting information file. DOI: 10.1107/S2056989018016663/rz5246Iasup4.cml

CCDC references: 1880871, 1880870

Additional supporting information:  crystallographic information; 3D view; checkCIF report

## Figures and Tables

**Table 1:** Hydrogen-bond geometry (Å, °) for **Ia** [Chem scheme1]

*D*—H⋯*A*	*D*—H	H⋯*A*	*D*⋯*A*	*D*—H⋯*A*
N1—H1*A*⋯O3^i^	0.89 (4)	1.83 (4)	2.707 (3)	167 (3)
N1—H1*B*⋯N2^ii^	0.79 (4)	2.21 (4)	2.907 (3)	148 (4)
N1—H1*C*⋯O4^iii^	0.87 (4)	1.88 (4)	2.732 (3)	167 (4)
O1—H1⋯N3	0.80 (5)	1.87 (6)	2.670 (3)	175 (5)

Symmetry codes: (i) 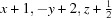; (ii) 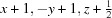; (iii) 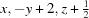.

**Table 2:** Hydrogen-bond geometry (Å, °) for **Ib** [Chem scheme1]

*D*—H⋯*A*	*D*—H	H⋯*A*	*D*⋯*A*	*D*—H⋯*A*
O1—H1⋯N3	0.82	1.87	2.677 (3)	167
N1—H1*A*⋯O3^i^	0.89	1.85	2.716 (3)	164
N1—H1*B*⋯N2^ii^	0.89	2.13	2.920 (3)	148
N1—H1*C*⋯O4^iii^	0.89	1.87	2.732 (3)	163

Symmetry codes: (i) 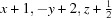; (ii) 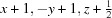; (iii) 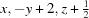.

**Table 3:** Experimental details

	150 K	293 K
Crystal data		
Chemical formula	C_7_H_8_NO_2_^+^·C_5_H_3_N_2_O_2_^−^	C_7_H_8_NO_2_^+^·C_5_H_3_N_2_O_2_^−^
*M*_r_	261.24	261.24
Crystal system, space group	Monoclinic, *P* *c*	Monoclinic, *P* *c*
*a*, *b*, *c* (Å)	5.95842 (16), 3.73769 (10), 25.5943 (6)	5.95233 (16), 3.80345 (11), 25.6879 (7)
β (°)	95.362 (2)	95.037 (2)
*V* (Å^3^)	567.51 (3)	579.31 (3)
*Z*	2	2
Radiation type	Mo *K*α	Mo *K*α
μ (mm^−1^)	0.12	0.12
Crystal size (mm)	0.24 × 0.19 × 0.18	0.24 × 0.19 × 0.18

Data collection		
Diffractometer	Rigaku Oxford Diffraxction Xcalibur Ruby Gemini ultra	Rigaku Oxford Diffraction Xcalibur Ruby Gemini ultra
Absorption correction	Multi-scan (*CrysAlis PRO*; Rigaku OD, 2018 ▸)	Multi-scan (*CrysAlis PRO*; Rigaku OD, 2018 ▸)
*T*_min_, *T*_max_	0.933, 1.000	0.822, 1.000
No. of measured, independent and observed [*I* > 2σ(*I*)] reflections	9340, 3426, 3244	8036, 2981, 2746
*R*_int_	0.024	0.033
(sin θ/λ)_max_ (Å^−1^)	0.727	0.694

Refinement		
*R*[*F* ^2^ > 2σ(*F* ^2^)], *wR*(*F* ^2^), *S*	0.044, 0.131, 1.13	0.049, 0.145, 1.11
No. of reflections	3426	2981
No. of parameters	216	174
No. of restraints	2	2
H-atom treatment	All H-atom parameters refined	H-atom parameters constrained
Δρ_max_, Δρ_min_ (e Å^−3^)	0.39, −0.28	0.29, −0.28

Computer programs: *CrysAlis PRO* (Rigaku OD, 2018 ▸), *SHELXT2014* (Sheldrick, 2015*a* ▸), *SHELXL2017* (Sheldrick, 2015*b* ▸) and *OLEX2* (Dolomanov *et al.*, 2009 ▸).

## supplementary crystallographic information

### 4-Carboxyanilinium pyrazine-2-carboxylate (Ia) Crystal data

**Table:**

C_7_H_8_NO_2_^+^·C_5_H_3_N_2_O_2_^−^	*F*(000) = 272
*M**_r_* = 261.24	*D*_x_ = 1.529 Mg m^−^^3^
Monoclinic, *P**c*	Mo *K*α radiation, λ = 0.71073 Å
*a* = 5.95842 (16) Å	Cell parameters from 5674 reflections
*b* = 3.73769 (10) Å	θ = 3.2–30.9°
*c* = 25.5943 (6) Å	µ = 0.12 mm^−^^1^
β = 95.362 (2)°	*T* = 150 K
*V* = 567.51 (3) Å^3^	Block, light colourless
*Z* = 2	0.24 × 0.19 × 0.18 mm

### 4-Carboxyanilinium pyrazine-2-carboxylate (Ia) Data collection

**Table:**

Rigaku Oxford Diffraction Xcalibur Ruby Gemini ultra diffractometer	3426 independent reflections
Radiation source: fine-focus sealed X-ray tube, Enhance (Mo) X-ray Source	3244 reflections with *I* > 2σ(*I*)
Graphite monochromator	*R*_int_ = 0.024
Detector resolution: 10.3457 pixels mm^-1^	θ_max_ = 31.1°, θ_min_ = 3.2°
ω scans	*h* = −8→8
Absorption correction: multi-scan (CrysAlis PRO; Rigaku OD, 2018)	*k* = −5→5
*T*_min_ = 0.933, *T*_max_ = 1.000	*l* = −36→36
9340 measured reflections	

### 4-Carboxyanilinium pyrazine-2-carboxylate (Ia) Refinement

**Table:**

Refinement on *F*^2^	2 restraints
Least-squares matrix: full	Hydrogen site location: difference Fourier map
*R*[*F*^2^ > 2σ(*F*^2^)] = 0.044	All H-atom parameters refined
*wR*(*F*^2^) = 0.131	*w* = 1/[σ^2^(*F*_o_^2^) + (0.0925*P*)^2^] where *P* = (*F*_o_^2^ + 2*F*_c_^2^)/3
*S* = 1.13	(Δ/σ)_max_ = 0.001
3426 reflections	Δρ_max_ = 0.39 e Å^−^^3^
216 parameters	Δρ_min_ = −0.28 e Å^−^^3^

### 4-Carboxyanilinium pyrazine-2-carboxylate (Ia) Special details

**Table:**

Geometry. All esds (except the esd in the dihedral angle between two l.s. planes) are estimated using the full covariance matrix. The cell esds are taken into account individually in the estimation of esds in distances, angles and torsion angles; correlations between esds in cell parameters are only used when they are defined by crystal symmetry. An approximate (isotropic) treatment of cell esds is used for estimating esds involving l.s. planes.

### 4-Carboxyanilinium pyrazine-2-carboxylate (Ia) Fractional atomic coordinates and isotropic or equivalent isotropic displacement parameters (Å^2^)

**Table:**

	*x*	*y*	*z*	*U*_iso_*/*U*_eq_	
O3	0.3138 (3)	0.3939 (5)	0.25476 (7)	0.0243 (4)	
O4	0.6224 (3)	0.5432 (6)	0.30735 (7)	0.0277 (4)	
O1	0.4407 (3)	0.5831 (6)	0.53444 (8)	0.0294 (4)	
O2	0.7570 (4)	0.7455 (6)	0.50022 (7)	0.0315 (5)	
N2	0.1012 (3)	0.1876 (5)	0.33842 (7)	0.0188 (4)	
N1	0.9340 (3)	1.2049 (5)	0.74469 (7)	0.0168 (3)	
N3	0.2855 (3)	0.3515 (6)	0.43916 (8)	0.0216 (4)	
C8	0.4243 (4)	0.4341 (6)	0.29860 (8)	0.0182 (4)	
C5	0.8626 (3)	1.0835 (6)	0.69173 (8)	0.0158 (4)	
C9	0.3040 (4)	0.3411 (6)	0.34657 (8)	0.0166 (4)	
C6	0.6482 (4)	0.9450 (6)	0.68152 (8)	0.0183 (4)	
C1	0.6445 (4)	0.7247 (6)	0.53737 (9)	0.0215 (4)	
C7	0.5784 (3)	0.8259 (6)	0.63124 (8)	0.0186 (4)	
C2	0.7220 (4)	0.8505 (6)	0.59139 (8)	0.0176 (4)	
C3	0.9376 (4)	0.9931 (7)	0.60225 (9)	0.0200 (4)	
C12	0.3970 (4)	0.4200 (6)	0.39723 (8)	0.0188 (4)	
C4	1.0095 (4)	1.1100 (6)	0.65252 (9)	0.0187 (4)	
C10	−0.0077 (4)	0.1148 (6)	0.38037 (9)	0.0208 (4)	
C11	0.0822 (4)	0.1982 (6)	0.43081 (9)	0.0210 (4)	
H7	0.437 (6)	0.717 (10)	0.6221 (13)	0.021 (8)*	
H10	−0.150 (8)	−0.003 (11)	0.3727 (18)	0.037 (10)*	
H4	1.156 (6)	1.214 (9)	0.6619 (14)	0.020 (8)*	
H3	1.033 (6)	1.003 (9)	0.5770 (15)	0.021 (8)*	
H12	0.550 (6)	0.517 (9)	0.4054 (14)	0.020 (8)*	
H1A	1.046 (7)	1.362 (10)	0.7450 (15)	0.028 (9)*	
H11	0.005 (6)	0.147 (10)	0.4628 (15)	0.027 (9)*	
H1B	0.978 (7)	1.037 (11)	0.7611 (17)	0.034 (10)*	
H6	0.553 (6)	0.919 (9)	0.7079 (14)	0.020 (7)*	
H1C	0.823 (7)	1.291 (9)	0.7600 (15)	0.026 (8)*	
H1	0.399 (8)	0.505 (14)	0.506 (2)	0.046 (12)*	

### 4-Carboxyanilinium pyrazine-2-carboxylate (Ia) Atomic displacement parameters (Å^2^)

**Table:**

	*U*^11^	*U*^22^	*U*^33^	*U*^12^	*U*^13^	*U*^23^
O3	0.0226 (8)	0.0359 (9)	0.0146 (7)	0.0076 (7)	0.0026 (6)	−0.0009 (7)
O4	0.0201 (8)	0.0422 (10)	0.0210 (8)	−0.0057 (7)	0.0034 (6)	0.0067 (7)
O1	0.0264 (9)	0.0470 (11)	0.0151 (8)	−0.0116 (8)	0.0028 (6)	−0.0063 (8)
O2	0.0337 (10)	0.0464 (11)	0.0157 (8)	−0.0122 (9)	0.0085 (7)	−0.0059 (7)
N2	0.0165 (8)	0.0229 (9)	0.0171 (8)	0.0001 (7)	0.0014 (6)	−0.0016 (7)
N1	0.0163 (8)	0.0204 (8)	0.0138 (8)	0.0004 (7)	0.0013 (6)	−0.0006 (6)
N3	0.0238 (10)	0.0272 (9)	0.0138 (8)	−0.0024 (7)	0.0021 (7)	−0.0009 (7)
C8	0.0174 (9)	0.0237 (10)	0.0140 (9)	0.0042 (8)	0.0042 (7)	0.0021 (7)
C5	0.0174 (9)	0.0183 (9)	0.0118 (8)	0.0014 (7)	0.0017 (7)	−0.0003 (7)
C9	0.0166 (9)	0.0196 (9)	0.0138 (9)	0.0021 (7)	0.0018 (7)	−0.0008 (7)
C6	0.0166 (9)	0.0243 (10)	0.0144 (9)	−0.0017 (7)	0.0036 (7)	0.0005 (7)
C1	0.0253 (11)	0.0242 (10)	0.0147 (9)	−0.0018 (8)	0.0005 (8)	−0.0011 (8)
C7	0.0172 (9)	0.0243 (10)	0.0142 (8)	−0.0033 (8)	0.0017 (7)	−0.0005 (7)
C2	0.0191 (9)	0.0216 (10)	0.0122 (8)	0.0001 (7)	0.0018 (7)	−0.0002 (7)
C3	0.0208 (10)	0.0271 (10)	0.0128 (9)	−0.0017 (8)	0.0057 (7)	−0.0019 (8)
C12	0.0161 (9)	0.0242 (10)	0.0161 (9)	−0.0029 (8)	0.0015 (7)	−0.0004 (8)
C4	0.0160 (9)	0.0243 (10)	0.0161 (9)	−0.0033 (7)	0.0034 (7)	−0.0015 (7)
C10	0.0183 (9)	0.0248 (10)	0.0193 (10)	−0.0035 (8)	0.0027 (7)	−0.0017 (8)
C11	0.0214 (10)	0.0255 (10)	0.0168 (10)	−0.0019 (8)	0.0050 (8)	0.0015 (8)

### 4-Carboxyanilinium pyrazine-2-carboxylate (Ia) Geometric parameters (Å, º)

**Table:**

O3—C8	1.256 (3)	C5—C4	1.395 (3)
O4—C8	1.249 (3)	C9—C12	1.393 (3)
O1—C1	1.320 (3)	C6—C7	1.388 (3)
O1—H1	0.80 (5)	C6—H6	0.93 (4)
O2—C1	1.216 (3)	C1—C2	1.492 (3)
N2—C9	1.336 (3)	C7—C2	1.394 (3)
N2—C10	1.334 (3)	C7—H7	0.95 (4)
N1—C5	1.455 (3)	C2—C3	1.394 (3)
N1—H1A	0.89 (4)	C3—C4	1.388 (3)
N1—H1B	0.79 (4)	C3—H3	0.90 (4)
N1—H1C	0.87 (4)	C12—H12	0.99 (4)
N3—C12	1.338 (3)	C4—H4	0.97 (4)
N3—C11	1.340 (3)	C10—C11	1.386 (3)
C8—C9	1.519 (3)	C10—H10	0.96 (5)
C5—C6	1.380 (3)	C11—H11	0.99 (4)
			
C1—O1—H1	114 (4)	O2—C1—C2	124.0 (2)
C10—N2—C9	117.57 (18)	C6—C7—C2	120.38 (19)
C5—N1—H1A	112 (2)	C6—C7—H7	123 (2)
C5—N1—H1B	108 (3)	C2—C7—H7	116 (2)
C5—N1—H1C	112 (2)	C7—C2—C1	119.9 (2)
H1A—N1—H1B	108 (4)	C7—C2—C3	119.75 (19)
H1A—N1—H1C	111 (3)	C3—C2—C1	120.30 (19)
H1B—N1—H1C	107 (4)	C2—C3—H3	120 (2)
C12—N3—C11	117.6 (2)	C4—C3—C2	120.2 (2)
O3—C8—C9	116.57 (19)	C4—C3—H3	120 (2)
O4—C8—O3	127.4 (2)	N3—C12—C9	121.5 (2)
O4—C8—C9	116.05 (19)	N3—C12—H12	115 (2)
C6—C5—N1	118.57 (18)	C9—C12—H12	124 (2)
C6—C5—C4	121.47 (19)	C5—C4—H4	118 (2)
C4—C5—N1	119.95 (19)	C3—C4—C5	119.0 (2)
N2—C9—C8	117.39 (18)	C3—C4—H4	123 (2)
N2—C9—C12	120.8 (2)	N2—C10—C11	122.0 (2)
C12—C9—C8	121.83 (19)	N2—C10—H10	115 (3)
C5—C6—C7	119.17 (19)	C11—C10—H10	123 (3)
C5—C6—H6	121 (2)	N3—C11—C10	120.7 (2)
C7—C6—H6	119 (2)	N3—C11—H11	116 (2)
O1—C1—C2	112.5 (2)	C10—C11—H11	124 (2)
O2—C1—O1	123.6 (2)		
			
O3—C8—C9—N2	7.2 (3)	C5—C6—C7—C2	−0.9 (3)
O3—C8—C9—C12	−171.1 (2)	C9—N2—C10—C11	0.9 (3)
O4—C8—C9—N2	−172.9 (2)	C6—C5—C4—C3	−0.2 (3)
O4—C8—C9—C12	8.7 (3)	C6—C7—C2—C1	−179.4 (2)
O1—C1—C2—C7	−2.9 (3)	C6—C7—C2—C3	0.5 (4)
O1—C1—C2—C3	177.2 (2)	C1—C2—C3—C4	−180.0 (2)
O2—C1—C2—C7	178.0 (2)	C7—C2—C3—C4	0.1 (3)
O2—C1—C2—C3	−1.9 (4)	C2—C3—C4—C5	−0.2 (3)
N2—C9—C12—N3	−1.3 (3)	C12—N3—C11—C10	0.0 (3)
N2—C10—C11—N3	−1.1 (4)	C4—C5—C6—C7	0.7 (3)
N1—C5—C6—C7	−179.6 (2)	C10—N2—C9—C8	−178.14 (19)
N1—C5—C4—C3	−179.9 (2)	C10—N2—C9—C12	0.2 (3)
C8—C9—C12—N3	177.0 (2)	C11—N3—C12—C9	1.1 (3)

### 4-Carboxyanilinium pyrazine-2-carboxylate (Ia) Hydrogen-bond geometry (Å, º)

**Table:**

*D*—H···*A*	*D*—H	H···*A*	*D*···*A*	*D*—H···*A*
N1—H1*A*···O3^i^	0.89 (4)	1.83 (4)	2.707 (3)	167 (3)
N1—H1*B*···N2^ii^	0.79 (4)	2.21 (4)	2.907 (3)	148 (4)
N1—H1*C*···O4^iii^	0.87 (4)	1.88 (4)	2.732 (3)	167 (4)
O1—H1···N3	0.80 (5)	1.87 (6)	2.670 (3)	175 (5)

Symmetry codes: (i) *x*+1, −*y*+2, *z*+1/2; (ii) *x*+1, −*y*+1, *z*+1/2; (iii) *x*, −*y*+2, *z*+1/2.

### 4-Carboxyanilinium pyrazine-2-carboxylate (Ib) Crystal data

**Table:**

C_7_H_8_NO_2_^+^·C_5_H_3_N_2_O_2_^−^	*F*(000) = 272
*M**_r_* = 261.24	*D*_x_ = 1.498 Mg m^−^^3^
Monoclinic, *P**c*	Mo *K*α radiation, λ = 0.71073 Å
*a* = 5.95233 (16) Å	Cell parameters from 4235 reflections
*b* = 3.80345 (11) Å	θ = 3.2–29.2°
*c* = 25.6879 (7) Å	µ = 0.12 mm^−^^1^
β = 95.037 (2)°	*T* = 293 K
*V* = 579.31 (3) Å^3^	Block, light colourless
*Z* = 2	0.24 × 0.19 × 0.18 mm

### 4-Carboxyanilinium pyrazine-2-carboxylate (Ib) Data collection

**Table:**

Rigaku Oxford Diffraction Xcalibur Ruby Gemini ultra diffractometer	2981 independent reflections
Radiation source: fine-focus sealed X-ray tube, Enhance (Mo) X-ray Source	2746 reflections with *I* > 2σ(*I*)
Graphite monochromator	*R*_int_ = 0.033
Detector resolution: 10.3457 pixels mm^-1^	θ_max_ = 29.6°, θ_min_ = 1.6°
ω scans	*h* = −7→8
Absorption correction: multi-scan (CrysAlis PRO; Rigaku OD, 2018)	*k* = −5→5
*T*_min_ = 0.822, *T*_max_ = 1.000	*l* = −35→33
8036 measured reflections	

### 4-Carboxyanilinium pyrazine-2-carboxylate (Ib) Refinement

**Table:**

Refinement on *F*^2^	2 restraints
Least-squares matrix: full	Hydrogen site location: inferred from neighbouring sites
*R*[*F*^2^ > 2σ(*F*^2^)] = 0.049	H-atom parameters constrained
*wR*(*F*^2^) = 0.145	*w* = 1/[σ^2^(*F*_o_^2^) + (0.0973*P*)^2^] where *P* = (*F*_o_^2^ + 2*F*_c_^2^)/3
*S* = 1.11	(Δ/σ)_max_ = 0.001
2981 reflections	Δρ_max_ = 0.29 e Å^−^^3^
174 parameters	Δρ_min_ = −0.28 e Å^−^^3^

### 4-Carboxyanilinium pyrazine-2-carboxylate (Ib) Special details

**Table:**

Geometry. All esds (except the esd in the dihedral angle between two l.s. planes) are estimated using the full covariance matrix. The cell esds are taken into account individually in the estimation of esds in distances, angles and torsion angles; correlations between esds in cell parameters are only used when they are defined by crystal symmetry. An approximate (isotropic) treatment of cell esds is used for estimating esds involving l.s. planes.

### 4-Carboxyanilinium pyrazine-2-carboxylate (Ib) Fractional atomic coordinates and isotropic or equivalent isotropic displacement parameters (Å^2^)

**Table:**

	*x*	*y*	*z*	*U*_iso_*/*U*_eq_	
O3	0.3114 (4)	0.4159 (6)	0.25493 (8)	0.0428 (6)	
O4	0.6195 (4)	0.5580 (8)	0.30677 (9)	0.0505 (6)	
O1	0.4431 (4)	0.5870 (8)	0.53404 (9)	0.0547 (7)	
H1	0.415003	0.501947	0.504859	0.082*	
O2	0.7585 (5)	0.7517 (8)	0.50073 (10)	0.0588 (7)	
N2	0.1020 (4)	0.2017 (6)	0.33869 (9)	0.0328 (5)	
N1	0.9323 (4)	1.1834 (6)	0.74503 (8)	0.0282 (5)	
H1A	1.042185	1.341470	0.744245	0.034*	
H1B	0.982136	0.999337	0.764065	0.034*	
H1C	0.815616	1.279554	0.759180	0.034*	
N3	0.2877 (4)	0.3635 (7)	0.43878 (10)	0.0383 (6)	
C8	0.4229 (4)	0.4514 (8)	0.29826 (10)	0.0314 (6)	
C5	0.8611 (4)	1.0681 (7)	0.69163 (9)	0.0262 (5)	
C9	0.3039 (4)	0.3555 (7)	0.34649 (10)	0.0273 (5)	
C6	0.6491 (4)	0.9282 (8)	0.68141 (10)	0.0312 (6)	
H6	0.552972	0.909327	0.707932	0.037*	
C1	0.6453 (5)	0.7272 (9)	0.53745 (10)	0.0365 (6)	
C7	0.5796 (4)	0.8159 (8)	0.63147 (11)	0.0319 (6)	
H7	0.436812	0.718844	0.624423	0.038*	
C2	0.7226 (4)	0.8473 (8)	0.59157 (10)	0.0301 (5)	
C3	0.9362 (5)	0.9920 (8)	0.60255 (11)	0.0356 (6)	
H3	1.032069	1.014408	0.576030	0.043*	
C12	0.3970 (5)	0.4333 (8)	0.39670 (11)	0.0335 (6)	
H12	0.538987	0.536543	0.401120	0.040*	
C4	1.0070 (4)	1.1027 (8)	0.65257 (11)	0.0330 (6)	
H4	1.149948	1.198795	0.659963	0.040*	
C10	−0.0051 (5)	0.1270 (8)	0.38076 (13)	0.0376 (6)	
H10	−0.144761	0.016045	0.376452	0.045*	
C11	0.0861 (5)	0.2102 (9)	0.43080 (12)	0.0390 (7)	
H11	0.005353	0.158345	0.459237	0.047*	

### 4-Carboxyanilinium pyrazine-2-carboxylate (Ib) Atomic displacement parameters (Å^2^)

**Table:**

	*U*^11^	*U*^22^	*U*^33^	*U*^12^	*U*^13^	*U*^23^
O3	0.0390 (12)	0.0655 (15)	0.0241 (10)	0.0140 (10)	0.0039 (8)	−0.0003 (9)
O4	0.0356 (12)	0.0830 (18)	0.0336 (12)	−0.0103 (11)	0.0068 (9)	0.0122 (11)
O1	0.0491 (13)	0.0908 (18)	0.0241 (10)	−0.0236 (13)	0.0034 (9)	−0.0131 (11)
O2	0.0618 (16)	0.0920 (19)	0.0244 (10)	−0.0238 (15)	0.0149 (10)	−0.0122 (12)
N2	0.0273 (10)	0.0424 (12)	0.0285 (11)	−0.0009 (9)	0.0011 (8)	−0.0052 (9)
N1	0.0288 (10)	0.0343 (11)	0.0211 (10)	0.0011 (8)	0.0007 (7)	−0.0007 (8)
N3	0.0429 (14)	0.0498 (14)	0.0221 (11)	−0.0049 (10)	0.0027 (9)	−0.0028 (10)
C8	0.0295 (12)	0.0427 (14)	0.0226 (12)	0.0078 (10)	0.0059 (9)	0.0036 (10)
C5	0.0292 (12)	0.0305 (13)	0.0190 (11)	0.0026 (9)	0.0022 (9)	0.0009 (9)
C9	0.0269 (12)	0.0331 (12)	0.0218 (11)	0.0028 (9)	0.0022 (9)	−0.0011 (9)
C6	0.0288 (12)	0.0441 (15)	0.0215 (12)	−0.0042 (10)	0.0067 (9)	0.0011 (10)
C1	0.0420 (15)	0.0458 (15)	0.0217 (12)	−0.0049 (12)	0.0033 (11)	−0.0035 (11)
C7	0.0278 (12)	0.0429 (14)	0.0253 (12)	−0.0056 (11)	0.0045 (10)	−0.0015 (10)
C2	0.0339 (13)	0.0363 (14)	0.0202 (11)	0.0010 (10)	0.0028 (9)	−0.0004 (9)
C3	0.0349 (14)	0.0514 (16)	0.0218 (12)	−0.0053 (11)	0.0092 (10)	−0.0024 (11)
C12	0.0287 (12)	0.0443 (16)	0.0274 (13)	−0.0059 (11)	0.0017 (10)	−0.0027 (11)
C4	0.0265 (12)	0.0441 (15)	0.0286 (13)	−0.0065 (10)	0.0042 (9)	−0.0035 (11)
C10	0.0303 (13)	0.0466 (16)	0.0364 (15)	−0.0082 (12)	0.0061 (10)	−0.0033 (12)
C11	0.0413 (16)	0.0484 (16)	0.0285 (14)	−0.0042 (12)	0.0109 (11)	−0.0001 (11)

### 4-Carboxyanilinium pyrazine-2-carboxylate (Ib) Geometric parameters (Å, º)

**Table:**

O3—C8	1.252 (3)	C5—C4	1.390 (4)
O4—C8	1.240 (4)	C9—C12	1.390 (4)
O1—H1	0.8200	C6—H6	0.9300
O1—C1	1.313 (4)	C6—C7	1.380 (4)
O2—C1	1.210 (4)	C1—C2	1.497 (4)
N2—C9	1.336 (4)	C7—H7	0.9300
N2—C10	1.332 (4)	C7—C2	1.394 (4)
N1—H1A	0.8900	C2—C3	1.391 (4)
N1—H1B	0.8900	C3—H3	0.9300
N1—H1C	0.8900	C3—C4	1.382 (4)
N1—C5	1.467 (3)	C12—H12	0.9300
N3—C12	1.336 (4)	C4—H4	0.9300
N3—C11	1.334 (4)	C10—H10	0.9300
C8—C9	1.524 (4)	C10—C11	1.388 (4)
C5—C6	1.374 (3)	C11—H11	0.9300
			
C1—O1—H1	109.5	O2—C1—C2	123.6 (3)
C10—N2—C9	117.4 (2)	C6—C7—H7	119.8
H1A—N1—H1B	109.5	C6—C7—C2	120.3 (2)
H1A—N1—H1C	109.5	C2—C7—H7	119.8
H1B—N1—H1C	109.5	C7—C2—C1	119.9 (2)
C5—N1—H1A	109.5	C3—C2—C1	120.6 (2)
C5—N1—H1B	109.5	C3—C2—C7	119.4 (2)
C5—N1—H1C	109.5	C2—C3—H3	119.8
C11—N3—C12	117.3 (3)	C4—C3—C2	120.5 (2)
O3—C8—C9	116.7 (2)	C4—C3—H3	119.8
O4—C8—O3	127.6 (3)	N3—C12—C9	121.7 (3)
O4—C8—C9	115.7 (2)	N3—C12—H12	119.1
C6—C5—N1	118.7 (2)	C9—C12—H12	119.1
C6—C5—C4	121.4 (2)	C5—C4—H4	120.5
C4—C5—N1	120.0 (2)	C3—C4—C5	118.9 (2)
N2—C9—C8	117.3 (2)	C3—C4—H4	120.5
N2—C9—C12	120.8 (2)	N2—C10—H10	119.1
C12—C9—C8	121.8 (2)	N2—C10—C11	121.8 (3)
C5—C6—H6	120.2	C11—C10—H10	119.1
C5—C6—C7	119.5 (2)	N3—C11—C10	121.0 (3)
C7—C6—H6	120.2	N3—C11—H11	119.5
O1—C1—C2	113.0 (2)	C10—C11—H11	119.5
O2—C1—O1	123.3 (3)		
			
O3—C8—C9—N2	8.0 (4)	C5—C6—C7—C2	−0.7 (4)
O3—C8—C9—C12	−170.1 (3)	C9—N2—C10—C11	1.1 (5)
O4—C8—C9—N2	−172.4 (3)	C6—C5—C4—C3	−0.3 (4)
O4—C8—C9—C12	9.5 (4)	C6—C7—C2—C1	−179.6 (3)
O1—C1—C2—C7	−2.3 (4)	C6—C7—C2—C3	0.2 (4)
O1—C1—C2—C3	177.9 (3)	C1—C2—C3—C4	180.0 (3)
O2—C1—C2—C7	178.7 (3)	C7—C2—C3—C4	0.2 (4)
O2—C1—C2—C3	−1.0 (5)	C2—C3—C4—C5	−0.2 (4)
N2—C9—C12—N3	−1.1 (5)	C12—N3—C11—C10	0.1 (5)
N2—C10—C11—N3	−1.2 (5)	C4—C5—C6—C7	0.7 (4)
N1—C5—C6—C7	−179.6 (3)	C10—N2—C9—C8	−178.1 (2)
N1—C5—C4—C3	180.0 (3)	C10—N2—C9—C12	0.0 (4)
C8—C9—C12—N3	176.9 (3)	C11—N3—C12—C9	1.1 (5)

### 4-Carboxyanilinium pyrazine-2-carboxylate (Ib) Hydrogen-bond geometry (Å, º)

**Table:**

*D*—H···*A*	*D*—H	H···*A*	*D*···*A*	*D*—H···*A*
O1—H1···N3	0.82	1.87	2.677 (3)	167
N1—H1*A*···O3^i^	0.89	1.85	2.716 (3)	164
N1—H1*B*···N2^ii^	0.89	2.13	2.920 (3)	148
N1—H1*C*···O4^iii^	0.89	1.87	2.732 (3)	163

Symmetry codes: (i) *x*+1, −*y*+2, *z*+1/2; (ii) *x*+1, −*y*+1, *z*+1/2; (iii) *x*, −*y*+2, *z*+1/2.
